# Evaluation of a species-specific C-reactive protein assay for the dog on the ABX Pentra 400 clinical chemistry analyzer

**DOI:** 10.1186/s12917-017-1065-9

**Published:** 2017-05-30

**Authors:** Sarah Hindenberg, Stefanie Klenner-Gastreich, Nicole Kneier, Sabine Zielinsky, Kris Gommeren, Natali Bauer, Andreas Moritz

**Affiliations:** 1 Department of Veterinary Clinical Sciences, Clinical Pathology and Clinical Pathophysiology, Justus-Liebig-University Giessen, 35392 Giessen, Germany; 2 Scil animal care company GmbH, 68519 Viernheim, Germany; 3 Department of Clinical Sciences, School of Veterinary Medicine, University of Liège, 4000 Liège, Belgium

**Keywords:** Acute phase protein, Canine, Method validation, Analyzer, Repeatability, Linearity, Total allowable error, Heparin plasma, Interference

## Abstract

**Background:**

A canine-specific immunoturbidimetric CRP assay, Gentian Canine CRP Immunoassay) with species-specific controls and calibrators was introduced and recently evaluated on the clinical chemistry analyzer Abbott Architect c4000 as well as on the Olympus AU600.

Aims of our study were 1) to independently evaluate the canine-specific CRP assay on the ABX Pentra 400 clinical chemistry analyzer in comparison to the previously validated human-based immunoturbidimetric assay (Randox Canine CRP assay) and 2) to assess the impact of different sample types (serum versus heparinized plasma) on the results.

Imprecision, accuracy, interference and the prozone effect were determined using samples from healthy and diseased dogs (*n* = 278). The Randox Canine CRP assay calibrated with canine specific control calibration material served as a reference method. Additionally, the impact of the sample type (serum and lithium heparin) was evaluated based on samples of healthy and diseased dogs (*n* = 49) in a second part of the study.

**Results:**

Linearity was present for CRP concentrations ranging from 4 to 281 mg/l. For clinically relevant CRP concentrations of 7–281 mg/l, recovery ranged between 90 and 105% and intra- and inter-assay CVs ranged between 0.68% - 12.12% and 0.88% - 7.84%, respectively. CV was thus lower than 12.16%, i.e. the desired CV% based on biological variation. Interference was not present up to a concentration of 5 g/l hemoglobin, 800 mg/l bilirubin and 10 g/l triglycerides. No prozone effect occurred up to 676 mg/l CRP. Method comparison study revealed a Spearman’s rank correlation coefficient of r_s_ = 0.98 and a mean constant bias of 5.2%. The sample type had a significant (*P* = 0.008) but clinically not relevant impact on the results (median CRP of 30.9 mg/l in lithium heparin plasma versus 31.4 mg/l in serum).

**Conclusions:**

The species-specific Gentian Canine CRP Immunoassay reliably detects canine CRP on the ABX Pentra 400 clinical chemistry analyzer whereby both serum and heparin plasma can be used. The quality criteria reached on the Abbott Architect c4000 and Olympus AU600 could be met.

## Background

C-reactive protein (CRP) is a major acute phase protein (APP) in the dog. As a part of innate immune response, APPs change their serum concentration in response to a systemic inflammation [1–4]. In contrast to classic inflammatory markers such as the white blood cell count, APPs react more rapidly and with a shorter half-life period [1, 5]. According to their kinetics in response to a pathological stimulus, positive APPs are classified as major, moderate and minor APPs. While major APPs show a 100- to 1000-fold increase within 24–48 h and decrease rapidly, moderate APPs react with a 5- to 10-fold increase within a period of 2–3 days and a slow decrease. In contrast, minor APPs react with a mild 1.5- to 2-fold increase [3]. Due to their marked, rapid increase, especially major APPs are sensitive diagnostic and prognostic measurands [6, 7] to monitor systemic inflammation. In dogs, an increase of CRP was shown in several conditions including infectious diseases [8–11], immune mediated diseases [12–14], neoplasias [12, 15, 16], and surgery [17].

A canine species-specific enzyme-linked immunosorbent assay (ELISA) [18] proved to be sensitive but too time-consuming to be used for routine measurements of canine CRP. First evaluation of immunoturbidimetric assays designed for the detection of human CRP showed unsatisfactory interspecies cross-reactivity between canine CRP and human CRP-antibodies [19], which was mainly attributed to a species-specific pattern of glycosylation of the CRP molecule [20, 21]. Later, an immunoturbidimetric assay with a reasonable cross-reactivity was evaluated [22].

In 2010, our group investigated three human-based immunoturbidimetric test systems in comparison to a species-specific ELISA [23]. However, application of human CRP reagents and heterologous control and calibration material may lead to unpredictable results with false low values in dog samples [22, 24]. The growing interest in standardizing and communicating the range of methods of APP measurement in veterinary species [2, 24, 25] led to the development of canine-specific assays with specific calibrators and controls. Meanwhile, purified canine CRP became commercially available and was used as calibrator in human CRP assays [24]. Canine specific controls as internal quality material still had to be prepared out of serum pools of healthy and diseased patients.

Recently, a species-specific immunoturbidimetric assay for canine CRP (Gentian Canine CRP Immunoassay, Gentian AS, Moss, Norway) became available and had been validated by Hillström et al. 2014 on the Abbott Architect c4000 (Abbott Park, IL, USA) in comparison to the Randox Canine CRP assay [26]. However, quality performance differences between various clinical chemistry analyzers have been demonstrated previously [27] making it difficult to both generalize results and compare studies. Muñoz-Prieto et al. investigated the same assay run on an Olympus AU600 analyzer but used the heterologous Olympus CRP assay as a reference method [28]. Apart from the analyzer, the sample material might also have an impact on assay performance. Acute phase proteins are commonly measured in serum, however, sometimes only either serum or lithium heparin plasma samples are submitted, when a clinical chemistry profile including CRP is requested. Unfortunately it is not always possible to collect a further comparable sample. It is therefore important to determine if both sample materials can be used interchangeably.

To the authors´ knowledge, the impact of the ABX Pentra 400 as a different analyzer on the performance of the Gentian CRP test as well as the effect of the sample material (serum versus heparin plasma) has not been evaluated so far. The aim of our study was thus to validate this assay with a different analyzer (i.e, Pentra 400 analyzer compared to the Abbott Architect c4000 and Olympus AU600 analyzer, respectively) using a similar methodology as the previous studies.

Our hypothesis was that the automated analyzer used has only a minor impact on the test performance; however, the sample material (heparin plasma versus serum) cannot be used interchangeably.

## Methods

### Study design

The present validation study was conducted between April 2013 and March 2016.

The study was structured into two parts: First, method validation was performed, including the evaluation of accuracy, recovery, precision, interference, prozone effect, and method comparison. The method comparison study was conducted according to recent recommendations [29], whereby the previously validated human-based immunoturbidimetric Randox Canine CRP assay (Randox Laboratories Ltd., Crumlin, UK) [23] served as the reference method. In the second part comparative measurements between lithium heparin plasma and serum samples were conducted to assess potential influence of an anticoagulant in order to simplify future routine diagnostics by allowing the application of both serum and heparin plasma samples.

### Measurement of C-reactive protein

Both the Gentian Canine CRP Immunoassay and the Randox Canine CRP assay were run on the ABX Pentra 400 clinical chemistry analyzer (Horiba ABX SAS, Montpellier, France).

#### Gentian canine CRP immunoassay

The canine-specific CRP assay (Gentian Canine CRP Immunoassay, Gentian AS, Moss, Norway) designed to be run on an automated analyzer is a quantitative immunoturbidimetric in-vitro diagnostic test using polyclonal chicken-derived canine-specific anti-CRP antibodies. The anti-CRP-immunoparticles aggregate with canine CRP and form complexes that can be measured with turbidimetric methods and are correlated with canine CRP concentrations by interpolation on a calibration curve. Calibration (Gentian Canine CRP Calibrator Kit, Gentian AS, Moss, Norway) and the measurement of canine-specific control material serving as internal quality control (Gentian Canine CRP Control Kit, Gentian AS, Moss, Norway) were performed daily.

#### Randox canine CRP assay

In our study, LOT 1303404 of Randox Canine CRP assay and - in contrast to the previous validation study [23] but in accordance with the previous validation study for the Gentian Canine CRP assay using the Randox Canine CRP assay as the reference method - a canine calibrator (Canine CRP Life Diagnostics, Inc., West Chester, USA) was used. Using canine calibration material for the human-based Randox Canine CRP assay, intra-assay CV ranged between 0.7% - 2.1% (unpublished data).

Measurements were performed as batch analysis at one day. Canine-specific calibration (Canine CRP Life Diagnostics, Inc., West Chester, USA) was done before the analyses were started while canine-specific control measurements (Canine CRP Reference Standard Biozol Diagnostica Vertrieb GmbH, Eching, Germany) were performed prior to the analyses and after each measurement of 60 samples.

### Method validation

To avoid preanalytical error due to numerous dilution steps, as it may occur by serially diluting a sample with very high CRP concentration to achieve a very low CRP concentration, linearity was evaluated in two dilution experiments with a partially overlapping CRP range: Linearity was first assessed by manual dilution of a canine serum specimen with markedly increased CRP concentration of 281.3 mg/l, achieving samples with 1.0, 0.8, 0.6, 0.4, 0.2, 0.1, 0.05, and 0.025 of the original CRP concentration. In addition, the diluted samples were used to calculate the recovery rate at eight different CRP levels.

To gain more information about the test performance at low CRP concentrations, linearity was also assessed by manual, stepwise dilution of pooled canine quality control material, whereby a normal and an abnormal control were mixed in equal parts (Gentian Canine CRP Control Kit, Gentian AS, Moss, Norway) so that a pooled sample with a mean CRP concentration of 66.5 mg/l was obtained that was then serially diluted to assess linearity at low CRP concentrations. For all dilution steps, double-distilled water was used as diluent to achieve samples with 1.0, 0.8, 0.6, 0.4, 0.2, 0.1, 0.05, 0.025 and 0.0125 of the original CRP concentration of the pooled samples. All samples were analyzed in triplicates in a single run. Intra-assay and inter-assay repeatability including between-run and between-day CV were determined by measurement of four canine serum samples with different CRP concentrations (A: 7.2 mg/l, B: 58.4 mg/l, C: 103.9 mg/l, D: 272.1 mg/l). Sample analysis was carried out in duplicates twice daily (morning and evening to assess between-run CV) over 5 days without recalibration. Sample order was changed randomly every run.As recommended for a verification of the limit of quantification [30], additional measurements were performed to evaluate intra-assay precision specifically for CRP values close to zero (A1: 2.3 mg/l, A2: 3.8 mg/l). Here, samples were analyzed 20 times in a single run without recalibration.

Interference was investigated by spiking aliquots of a canine serum sample of 35.5 mg/l CRP with 800 mg/l bilirubin (Bilirubin, Sigma-Aldrich Co. LLC., St. Louis, Missouri, USA), 5 g/l hemoglobin (Hemoglobin human, Sigma-Aldrich Co. LLC., St. Louis, Missouri, USA) or 10 g/l 20% soy bean emulsion (Intralipid 20%, Fresenius Kabi Canada, Ontario, Canada) before analyzing them in triplicates in random order.

A stock solution containing bilirubin in a concentration of 20 g/l was prepared by diluting 20 mg bilirubin in 1 ml of 0.1 M NaOH. Then, 0.1 ml of the product was added to 2.4 ml non-spiked serum sample to achieve a bilirubin level of 800 mg/l.Lyophilized human hemoglobin was dissolved in 0.09% NaCl (1 part in 10 parts of NaCl) so that a stock solution containing 100 g/l hemoglobin was obtained and 0.125 ml of the solution were added to 2.375 ml non-spiked serum sample to receive a hemoglobin concentration of 5 g/l.To assess the impact of lipemia on results, 0.125 ml of Intralipid were added to 2.375 ml of non-spiked serum sample resulting in concentration of soya bean oil of 10 g/l.

The samples were compared to serum aliquots spiked with an equal volume of either 100 mM NaOH (in case of bilirubin), 0.09% NaCl (hemoglobin) or pure water (in case of Intralipid). Antigen overload may provoke falsely low CRP measurements. This so called prozone effect was evaluated by spiking a serum sample containing a very low CRP concentration with purified canine CRP (Dog C-Reactive Protein, Life Diagnostics, Inc., West-Chester, USA) until a CRP concentration of 1069.5 mg/l was obtained. The spiked sample was subsequently diluted with 0.9% NaCl so that final concentrations of 455 mg/l, 676 mg/l, and 890 mg/l were achieved. Samples were measured in duplicates in a single run.

#### Method comparison study

In the method comparison study, the Gentian Canine CRP Immunoassay was compared to the previously validated human-based immunoturbidimetric Randox Canine CRP assay.

Overall, 278 serum samples of healthy and diseased dogs presented at the Department of Clinical Sciences, School of Veterinary Medicine, University of Liège, Liège, Belgium, were analyzed with both immunoturbidimetric assays. The samples have already been used for a previous study in which inflammatory cytokines and CRP were investigated in canine systemic inflammatory response syndrome patients [31]. In our study, residual sample material was used.

Samples were collected between January and August 2010, stored at −80 °C and shipped frozen to the Department of Veterinary Clinical Sciences, Clinical Pathology and Clinical Pathophysiology, Justus-Liebig-University Giessen, Germany, and stored again at −80 °C for 4 weeks until batch-analysis was performed.

#### Effect of sample material

To assess the impact of an anticoagulant (lithium heparin plasma versus serum sample) on the CRP measurements, blood samples of 49 healthy and diseased dogs presented at the Clinic for Small Animals, Faculty of Veterinary Medicine, Justus-Liebig University, Giessen, Germany, were obtained simultaneously by venipuncture with a sterile disposable cannula into tubes (1.3 ml) containing lithium heparin as anticoagulant and tubes without additives.

Inclusion criterion was that both a heparinized blood sample and a serum sample were taken for diagnostic purposes. Between March and April 2014, samples were analyzed in matched pairs within the first hour after blood collection. Lithium heparin anticoagulated samples were centrifuged for 1 min with 8944 g without delay, while samples without additives were allowed to clot for at least 10 min after receipt in the laboratory. Intra-assay repeatability for measurement of CRP in heparinized plasma was first assessed for ten consecutive measurements with samples from three dogs with low, moderate and high CRP values (10, 53, 77 mg/l respectively). Additionally, the LoQ of the CRP assay determined for serum (A1, A2, Table 1) was verified for heparinized plasma as well, whereby both the heparinized and the serum samples were taken from the same dogs with CRP values close to zero and analyzed 20 times.

**Table 1:** Precision of CRP determination in pooled serum samples with CRP concentrations below a clinical decision limit (A1 - A2) as well as pooled serum samples with clinically relevant CRP concentrations

		Intra-assay CV		Inter-assay CV			
				Between run CV		Between day CV	
Level	Mean CRP concentration (mg/l)	SD (mg/l)	CV%	SD (mg/l)	CV%	SD (mg/l)	CV%
A1	2.3	0.72	**31.1**	n.d.	n.d.	n.d.	n.d.
A2	3.8	0.35	9.3	n.d.	n.d.	n.d.	n.d.
A	7.2	0.87	12.1	0.56	7.8	0.44	6.1
B	58.4	1.08	1.9	1.25	2.1	2.7	4.6
C	103.9	0.88	0.9	0.91	0.9	1.9	1.8
D	272.1	1.85	0.7	4.98	1.8	2.8	1.1

A1, A2: CVs for intra-assay imprecision of two canine serum pools analyzed 20 times in a single run without recalibration.

A – D: Inter- and intra-assay CVs of 6 canine serum pools analyzed in duplicates twice daily over 5 days.

CVs not fulfilling the quality specifications i.e., are higher than 12.2% [32] are marked in bold letters.Abbreviations: n.d. = not done, SD = standard deviation; CV = coefficient of variation.

#### Statistical analysis

Statistical analyses were carried out using statistical software packages (MedCalc, software version 16.2.1; Ostend, Belgium and GraphPad Prism 6 Software, GraphPad Software, Inc., La Jolla, USA).

#### Method validation study

Limits of acceptance were set according to Total allowable error (TE) guidelines of the American college of Veterinary Clinical Pathology (ASVCP) [32]. Although no definite TE for CRP was recommended by the workgroup, an optimal and desired coefficient of variation (CV), bias, and TE based on biologic variation was given in the addendum of the ASVCP guidelines: The optimal / desired allowable imprecision (CV_opt_/CV_des_) were reported to be 6.08% and 12.16% respectively, the optimal and desired allowable bias (bias_opt_/bias_des_) were 4.76% / 9.52% and the optimal / desired total allowable error (TE_opt_/TE_des_) were 14.79% / 29.58%, respectively [32]. Here, numbers rounded to one decimal place are used.

Descriptive statistics were performed to calculate arithmetic means, standard deviations (SD), and CV. All data were evaluated for normal distribution using the Shapiro-Wilk Test.

To evaluate the linearity under dilution of the assay, mean results of measured values were plotted against theoretical CRP concentration. The ‘best fit’ line was visually inspected for linearity. In addition, recovery after dilution was calculated by subtracting the measured CRP result from the calculated (expected) CRP concentration according to the following formula:

Recovery % = $$ \frac{measuredconcentration- expected concentration}{expectedconcentration} x100. $$

Acceptance criteria for recovery rates were set at 80–120% for samples as recommended previously for validation of immunoassays [33, 34]. The intra-assay CVs were calculated as follows based on mean and standard deviation (SD):

CV % = $$ \frac{SD}{Mean} x100 $$

For the interference study, non-spiked samples (control) and samples spiked with the interfering substances (test) were measured in triplicates. Observed interference effect (d_obs)_ was computed as the % bias between the means of the test and control specimens:

d_obs_ %$$ =\frac{meantest- mean control}{meancontrol} x100 $$

In accordance with the literature, bias between control and test sample was considered acceptable if the bias for the interfering substance (i.e., d_obs_ %) was smaller than the allowable TE [35], i.e. 29.6% (TE_des_), and 14.8% (TE_opt_) for canine CRP [32].

#### Method comparison study

Data of the method comparison experiment were analyzed with Spearman’s rank correlation and Passing & Bablok regression analysis. Bland-Altman difference plot was performed to investigate % bias. In addition, the total observed error TE_obs_ was calculated to judge acceptability according to the quality specifications recommended by the American Society for Veterinary Clinical Pathology (ASVCP) [32]. TE_obs_ was calculated using following formula:

TE_obs_ = bias % + 2CV%.

#### Effect of sample material

Comparative measurements of plasma samples: Normality was assessed with a Shapiro Wilk test. Since data were not normally distributed, Wilcoxon signed-rank test was performed to assess potential differences between the methods. For method validation, Spearman’s rank correlation as well as Passing-Bablok regression and Bland-Altman analysis were performed.

## Results

### Method validation study

Results of the precision study are shown in Table 1. For clinically relevant CRP concentrations ranging between 7.2 and 272.1 mg/l (concentrations A-D), both intra- and inter-assay CVs fulfilled the quality specifications, i.e., the CVs did not exceed 12.2% [32]. Intra-assay imprecision <2% was present for clinically relevant CRP-ranges >40 mg/l. Only for CRP concentrations of 2.5 mg/l, a CV markedly exceeding the desired CV was noted, so that the LoQ was set above the lowest concentrations still fulfilling the quality specifications i.e., 3.8 mg/l (≈ 4 mg/l). Evaluation of inter-assay precision showed slightly higher inter-assay CVs of 0.88–7.84% than intra-assay CVs.

**Fig. 1:**
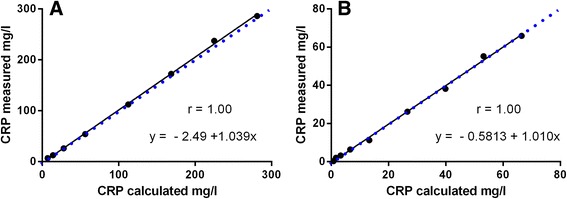
Linearity of CRP determination at a high (**a**) and low concentration range (**b**). (**a**) Linearity under dilution for measurement of a canine serum sample originally containing 281.3 mg/l CRP. A serial dilution was performed to achieve 8 different CRP concentrations, i.e., 1.0, 0.8, 0.6, 0.4, 0.2, 0.1, 0.05, 0.025 parts of the original concentration (**b**) Linearity range of CRP determination for a canine serum sample containing 66.5 mg/l CRP. A serial dilution was performed to achieve 9 different CRP concentrations i.e., 1.0, 0.8, 0.6, 0.4, 0.2, 0.1, 0.05, 0.025, 0.0125 parts of the original concentration

The results of the linearity study for higher and lower concentration ranges are demonstrated in Fig. 1, Table 2, and Table 3. As shown in Fig. 1, there was an excellent correlation between expected and measured values for both concentrations ranges. Considering the LoQ, linearity under dilution of the Gentian Canine CRP Immunoassay was present in a range of 4–281 mg/l for serially diluted canine specimens (Figure 1). Recovery rate ranged between 88.9–105.4% at expected CRP values of 7.0–281.3 mg/l (Table 2) and thus fulfilling the quality requirements. For CRP concentrations ranging between 0.8 mg/l and 66.5 mg/l (Table 3), however, quality requirements were not fulfilled for the lowest CRP concentrations of 0.8 mg/l and 1.7 mg/l, respectively.

**Table 2:** Linearity and recovery rates of CRP measurements in a clinically relevant range of 7.0–281.3 mg/l after serial dilution of a canine serum sample containing 281.3 mg/l CRP

Dilution Factor	Expected concentration [mg/l]	Mean measured concentration[mg/l]	Recovery [%]	Bias[%]	% bias < TE_des_ (29.6%)	% bias < TE_opt_ (14.8%)
0.025	7.0	6.9	97.6	−2.4	Yes	Yes
0.05	14.1	12.5	88.9	−11.1	Yes	Yes
0.1	28.1	26.1	92.9	−7.1	Yes	Yes
0.2	56.3	54.1	96.1	−3.9	Yes	Yes
0.4	112.5	112.3	99.8	−0.2	Yes	Yes
0.6	168.8	172.3	102.1	2.2	Yes	Yes
0.8	225.0	237.3	105.4	5.4	Yes	Yes
1	281.3	286.2	101.7	1.7	Yes	Yes

Recovery rates not fulfilling the quality specifications of 80–120% are marked in bold letters as well as % bias between expected and measured mean CRP concentration exceeding the desired (TE_des_) and optimal total allowable error (TE_opt_) reported previously [32].

**Table 3:** Linearity and recovery rates of CRP measurements at a lower concentration range of 0.8–66.5 mg/l obtained after serial dilution of a canine serum sample containing 66.5 mg/l CRP

Dilution Factor	Expected concentration [mg/l]	Mean measured concentration[mg/l]	Recovery [%]	Bias[%]	% bias < TE_des_ (29.6%)	% bias < TE_opt_ (14.8%)
0.0125	0.8	0.3	**36.1**	**−63.9**	**No**	**No**
0.025	1.7	2.0	**122.5**	22.5	Yes	**No**
0.05	3.3	3.3	98.4	−1.6	Yes	Yes
0.1	6.7	6.4	95.7	−4.3	Yes	Yes
0.2	13.3	11.2	84.3	−15.7	Yes	Yes
0.4	26.6	26.2	98.6	−1.4	Yes	Yes
0.6	39.9	38.1	95.6	−4.4	Yes	Yes
0.8	53.2	55.3	103.9	3.9	Yes	Yes
1	66.5	65.9	99.2	−0.8	Yes	Yes

For quality specifications regarding recovery rate and % bias, see Table 2.

Overall, no interference was detectable up to a concentration of 5 g/l hemoglobin, 800 mg/l bilirubin and 10 g/l soy bean oil (Table 4). Mean absolute bias between control and spiked test samples was 0.1 mg/l, 0.6 mg/l and 1.6 mg/l respectively. At a clinically relevant CRP concentration of about 30 mg/l, the systematic errors are lower than TE_opt_ for all interfering substances. No prozone effect was present up to a concentration of 676 mg/l CRP as CRP values were measured correctly: The recovery rates for high CRP concentrations were 130% for 455 mg/l, 121% for 676 mg/l and 28% for 890 mg/l, respectively.

**Table 4:** Interference effects of hemoglobin, bilirubin and lipid (soya bean oil) on CRP measurement performed with the new automated species-specific immunoturbidimetric assay

Interferent	CRP_control_ [mg/l] ± SD	CRP_test_ [mg/l] ± SD	Mean bias [mg/l]	% bias	% bias < TE_des_ (29.6%)	% bias < TE_opt_ (14.8%)
Hemoglobin 5 g/l	33.7 ± 0.7	34.3 ± 1	0.57	1.7	Yes	Yes
Bilirubin 800 mg/l	34.8 ± 0.6	34.9 ± 1	0.13	0.37	Yes	Yes
Soy bean emulsion 10 g/l	32.7 ± 0.2	34.4 ± 1	1.63	5.0	Yes	Yes

Test samples (CRP_test_) with a mean CRP value of 35.5 mg/l spiked with the interfering substances were measured in triplicates and compared to control samples (CRP_control_) spiked with equal amounts of the diluent used in the test sample. %Bias for the interfering substance was considered acceptable when it was < desired total allowable error (TE_des_) and excellent when it was < optimal total allowable error (TE_opt_) reported previously [32].

### Method comparison study

**Fig. 2:**
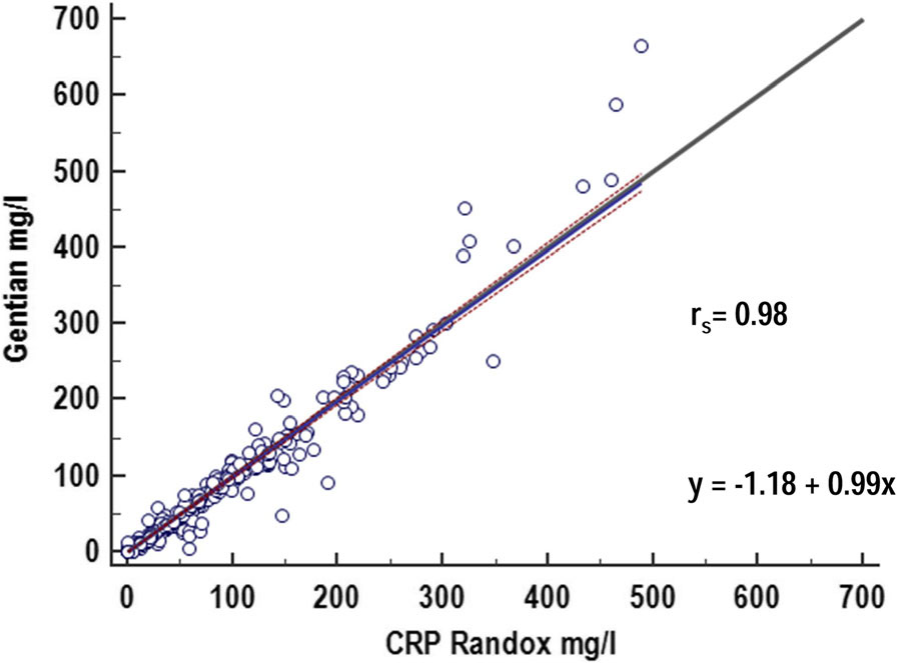
Passing-Bablok regression analysis for canine C-reactive protein determined in canine serum samples by use of a species-specific immunoturbidimetric assay (Gentian Canine CRP Immunoassay) in comparison to a previously validated human based immunoturbidimetric test system (Randox Canine CRP assay) run with a dog calibrator. The solid blue line illustrates the regression equation with its 95%-confidence intervals (brown dotted line). The thin solid grey line represents the identity line consistent with a perfect correlation of the two methods

**Fig. 3:**
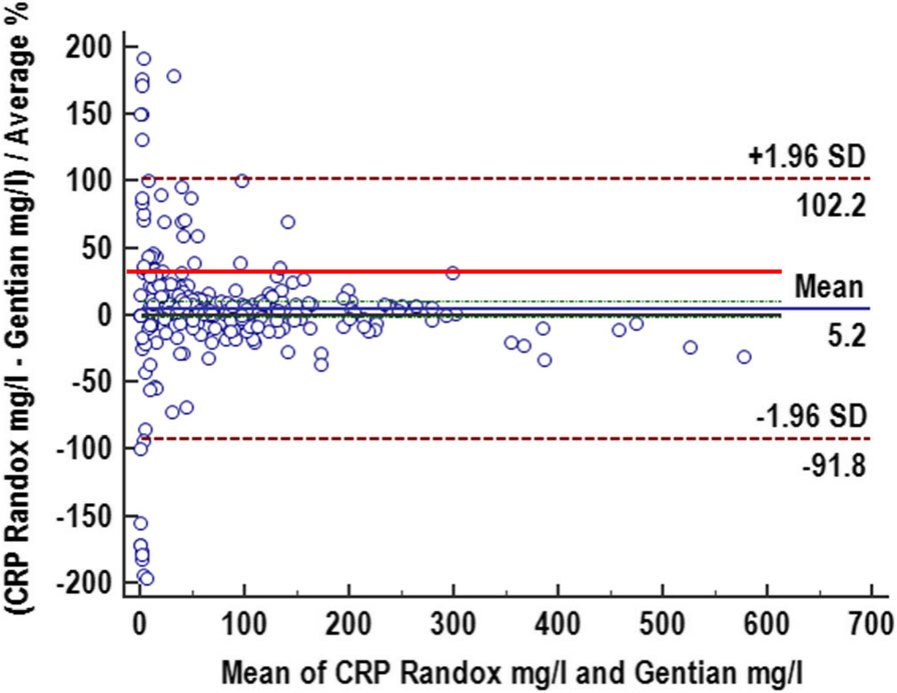
Bland-Altman difference plot for canine C-reactive protein measured in canine serum samples with a new species-specific immunoturbidimetric assay (Gentian Canine CRP Immunoassay) in comparison to a validated human based test system (Randox Canine CRP assay). The solid blue line demonstrates the mean % bias, the thin solid grey line is consistent with the identity line. The dashed brown lines show the limits of agreement, which are defined as the mean difference plus and minus 1.96 times the standard deviation (SD). The solid red line indicates the desired total allowable error (TE_des_) of 29.6% [32]. A small bias of 5.2% with a confidence interval of 95% (green dotted lines) is present

As demonstrated in Fig. 2, there was an excellent correlation (r_s_ = 0.98) between the results obtained with the species specific canine CRP test and the reference method. Passing-Bablok regression equation revealed small constant and proportional errors reflected by an intercept of −1.18 (with 95% confidence intervals (CI) of −2.07 to −0.43 mg/l) and a slope of 0.99 (95% CI 0.97 to 1.08) (Figure 2). Bland-Altman analysis revealed a mean constant bias of 5.2% (Figure 3). Taking the bias and the inter-assay CV at the 4 different CRP concentrations (A-D, Table 1) under consideration, TE_obs_ was calculated and results are shown in Table 5. TE_obs_ ranged from 20.9% at low levels to 7.0–9.5% at higher levels B-D (table 5). Overall, TE_obs_ was < TE_des_ of 29.6% for all CRP concentration levels and even < TE_opt_ of 14.8% for the clinically relevant concentration levels B-D [32].

**Table 5:** Observed total allowable error (TE_obs_ %) calculated at four different CRP levels taking the coefficient of variation (CV) and the bias of 5.2% derived from the method validation study under consideration

Level	A	B	C	D
CRP concentration mg/l	7.2	58.8	103.9	272.1
CV _Between-run_ %	7.84	2.14	0.88	1.83
TE_obs_ %	**20.9**	9.5	7.0	8.9
TE_obs_ < TE_des_	Yes	Yes	Yes	Yes
TE_obs_ < TE_opt_	**No**	Yes	Yes	Yes

TE_obs_ was < desired total error (TE_des_) of 29.6% [32] for all CRP concentration levels. TE_obs_ % results exceeding the optimal TE (TE_opt_) of 14.8% [32] are marked in bold letters.

### Effect of sample material

**Fig. 4:**
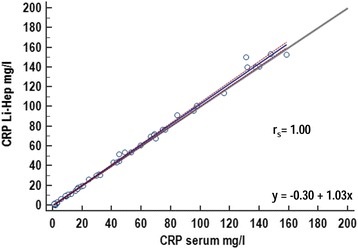
Passing-Bablok regression analysis detailing the comparison between results of canine C-reactive protein (CRP) determined in either serum samples or lithium heparin samples (Li-Hep) with the Gentian Canine CRP Immunoassay. The solid blue line illustrates the regression with its 95%-confidence intervals (brown dotted line). The diagonal grey line is consistent with the identity line

**Fig. 5:**
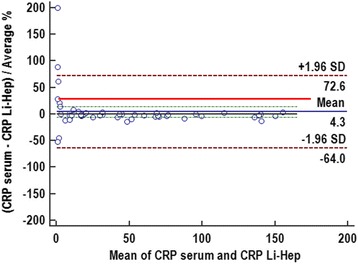
Bland-Altman difference plot for canine C-reactive protein (CRP) measured in canine serum samples and canine lithium heparin (Li-Hep) samples with a Gentian Canine CRP Immunoassay. The black line is consistent with the zero line. The blue line indicates the mean bias and its 95%-confidence interval (green dotted line). The dotted brown line is consistent with the ±1.96 standard deviation (SD) of the mean absolute bias indicating the limits of agreement. The red line indicates the desired maximum total allowable error (TE_des_) of 29.6% [32] for measurement of canine CRP

Spearman’s rank correlation coefficient revealed an excellent correlation between CRP measurements obtained in serum and heparin plasma samples (r_s_ = 0.995). There was a significant (*P* = 0.008), but clinically not relevant difference between the median CRP results obtained for both sample types (30.9 mg/l for lithium heparin plasma versus 31.4 mg/l for serum samples).Passing-Bablok regression equation showed a small constant error with an intercept of 0.29 and a small proportional error with a slope of 0.97 (Figure 4). Bland-Altman difference plot (Figure 5) revealed a mean bias of 4.3% between results obtained with both sample types.

## Discussion

Overall, the Gentian Canine CRP Immunoassay accurately and precisely detects canine CRP similar to the previously used human-based immunoturbidimetric method on the ABX Pentra 400 clinical chemistry analyzer.

Comparisons of results of the current and previous studies evaluating the Gentian CRP test for different analyzers are shown in Table 6. As seen in the table, comparable results have been obtained for the majority of quality parameters demonstrated on the Abbott Architect c4000 [26] so that performance quality could be also confirmed for the ABX Pentra 400. There are some small differences that have the potential to be clinically relevant including a different CRP concentration at which a prozone effect occurs, a slightly different LoQ and a different linearity range (table 6). The evaluation on the Olympus AU600 revealed a LoQ lying between the values of the other two studies (table 6). Linearity and prozone effect were not investigated in comparably high ranges as performed in the other studies. It remains unclear if the differences between the studies are due to pre-analytic or analytic factors. As such, evaluation of these discrepant quality parameters for different types of analyzer across laboratories is recommended. The main discrepancy between the current and the study by Hillström et al. is the CRP concentration at which a prozone effect is seen which is markedly higher (1200 mg/l) in the previous than in the current study (676 mg/l). Muñoz-Prieto et al. mentioned the prozone effect to occur above a CRP level of 400 mg/l but did not evaluate higher levels [28]. The term prozone effect describes the event of false low values of the analyte in immunoassays due to an excess of the analyte and is based on the saturation curve of the antibody binding capacity for antigen. The high number of analyte particles inhibits the formation of antigen-antibody complexes as all binding sites of the antibody are already occupied by antigen. This mechanism can lead to a false low signal detected by turbidimetry [36]. Regarding the origin of the markedly different prozone effect observed here and in the previous study, pre-analytic factors such as pipetting errors are considered an unlikely cause. However, analytical factors including analyzer performance, variations in test application or differences between variable batches of the test are a likely explanation for the discrepant prozone effect between the studies. Marked batch-specific differences in test performance due to manufacturing tolerances have been previously observed for the human Randox CRP assay when applied for canine specimens [23, 24]. However, the marked variation between different batches of the CRP assay was mainly caused by the highly variable cross-reactivity between anti-human-CRP antibodies applied in the test and canine CRP. In a canine species-specific CRP test, such high variation is unlikely, however, differences between various batches have to be evaluated in future studies. Another possible explanation would be a variation between different batches of calibrators which could influence the calibration curve in detail and therefore cause small differences in CRP values reported. It is a limitation of this study that it was not possible to directly compare the performance of both analyzers using the same batch of test reagents, calibrators and samples to exclude batch related variances. The instrument settings of the different analyzers used may contribute to a variation of the CRP-concentration causing a prozone effect. If the recommended standard instrument settings [37] are used, however, no major differences between the settings can be found. There is no pre-dilution of the sample by both analyzers which could potentially influence a prozone effect. Moreover, minor differences between the analyzers such as a minimally different measuring wave length (600 nm for the ABX Pentra 400, 604 nm for Abbott Architect c4000) are unlikely to have a major impact on the occurrence of a prozone effect.

**Table 6:** Comparison of observed quality parameters for the Gentian Canine CRP Immunoassay run on three different analyzers

Quality parameters Gentian CRP	ABX Pentra 400	Abbott Architect c4000^a^	Olympus AU600^b^
Limit of quantification (mg/l)	4.0^c^	6,8^d^	5.4^e^
Linearity (mg/l)	4–281	6.8–1201	~5–100
Recovery (%)	90–105	116–123	105–118
No prozone effect (mg/l)	≤ 676	≤ 1200	≤ 400
Precision			
CRP range (mg/l):	< 270; > 25	< 270; > 25	≤ 100, ≥ 50
*Intra-assay CV (%)*	0.7–1.9	0.5–1.7	1.0–1.3
*Inter-assay CV (%)*			
- Between run CV (%)	0.9–2.1	0.0–0.3	n.d.
- Between day CV (%)	1.1–4.6	1.1–1.9	4.1–4.7
No Interference up to			
- Hemoglobin (g/l)	5	10	n.d.
- Bilirubin (mg/l)	800	n.d.	n.d.
- Triglycerides (g/l)	10	10	n.d.
Method comparison			
Reference method	Randox Canine CRP assay	Randox Canine CRP assay	Olympus CRP
- r_s_	0.98	0.995	0.96
- intercept	-1.18	7.3	n.d.
- slope	0.99	0.92	n.d.
- mean constant bias (%)	5.2	n.d.	41.0–70.5^f^
- TE_obs_ (%) (~CRP 7–60 mg/l)	9.5–20.9	n.d.	50.4–82.6 ^f^
(~CRP > 100 mg/l)	7.0–8.9	n.d.	n.d
Sample type comparison	small impact	n.d.	n.d.

The assay was evaluated independently on the ABX Pentra 400 and compared to the data of the previous validation on the Abbott Architect c4000 and on the Olympus AU600.

Abbreviations: *n.d*. not done, *CV* coefficient of variation, *TE*_*obs*_ observed total allowable error

^a^(Hillström et al. 2014) ^b^(Muñoz-Prieto et al.2017)^c^based on the desirable CV < 12.16%, ^d^based on desirable TE < 29.58%; both published in the addendum of the Total allowable error (TE) guidelines of the American College of Veterinary Clinical Pathology [32], ^e^based on CV < 20% (Escribano et al. 2012). ^f^ calculated based on the data of healthy and diseased dogs evaluated by Muñoz-Prieto et al.: healthy dogs: $bias\%=\frac{median\  control- median\  test}{mean\  of\  the\  medians}=\frac{2.82\frac{mg}{l}-1.35\frac{mg}{l}}{\left(2.82-1.35\right):2} x100\%\approx 70.5\%$; TE_obs_ = bias % + 2CV% = 70.5% + 2 × 6.05% = 82.6%

dogs with inflammatory conditions:$bias\%=\frac{73.7\frac{mg}{l}-48.6\frac{mg}{l}}{\left(73.7+48.6\right):2} x100\%\approx 41.0\%$;TE_obs_ = 41.0% + 2 × 4.70% = 50.4%

The use of latex particles reduces prozone effects [36] as well as an endpoint measurement instead of a kinetic analysis, but both factors were constant here as the same assay was used on both analyzers.

In contrast, a different reagent volume and ratio of reagent to sample volume (270 μl of reagent 1, 75 μl of reagent 2, 3 μl sample volume for the ABX Pentra 400; 270 μl of reagent 1, 70 μl of reagent 2, 2 μl sample volume for Abbott Architect c4000) may be a possible explanation for different CRP concentrations at which a prozone effect occurs but it remains questionable if it is the only explanation for the major differences between prozone effects observed in the different studies. Although CRP concentrations >680 mg/l are extremely rare, septic dogs have been occasionally shown to have high CRP ranges of up to 632 mg/l [6] and in dogs with snake envenomation even CRP concentrations above 900 mg/l were detected [38].As the prozone effect was shown to occur above a CRP concentration of 680 mg/l, the issue of false low results in patients with these rarely occurring extremely high CRP values is a consideration. A correlation with other clinical and laboratory parameters is therefore mandatory to detect samples needing a pre-dilution before measurement.

The LoQ determined in the current study was slightly lower than the LoQ determined in previous validation studies [26, 28] (Table 6). In the current study, the LoQ was derived solely from the replication experiment, i.e. the quality goal was based on the CV_des_ published in the addendum of the allowable total error guidelines of the ASVCP [32] that had to be <12.16%. Muñoz-Prieto et al. used a higher CV of <20% as decision criterion [28]. The LoQ in the previous study by Hillström et al. was obtained from the linearity experiment taking both the CV and the bias between expected and measured values into consideration, i.e. the quality goal was based on the TE_des_ also published in the ACVCP guidelines. While both approaches are justified, it has to be considered that using the TE_des_ - and thus both bias and CV - is a more stringent approach than just applying CV_des_ and is therefore the most likely explanation for the LoQ set at a higher CRP concentration than in the current study (6.8 mg/l and 4.0 mg/l, Table 6). When regarding the results published previously, a SD of 0.39 mg/l was observed at a CRP concentration of 6.8 mg/l, consistent with a CV of 5.5% which would have fulfilled the quality goal of the current study. While a similar study design would have been preferable to allow an exact comparison between both analyzers and thus the true analyzer-dependent effect, both studies have been planned independently from each other in overlapping periods of time.

Possible contributing factors to differences between the studies might be also pre-analytical errors, especially due to pipetting as well as the impact of the analyzer. Also the LoQ determined by Muñoz-Prieto et al. is slightly higher (Table 6) which may be due to preanalytical or analyzer dependent conditions or influenced by the exact CRP levels used to evaluate the limit. However, the differences in LoQs observed in all three studies are rather academic in nature than of true clinical relevance as the clinical decision limit to differentiate between healthy dogs or dogs with and without systemic inflammation was 16.8 mg/l [38] and thus well above the LoQs found here and in the previous investigation.

For CRP concentrations above the LoQ of 4 mg/l, intra-assay and inter-assay CVs ranging between 0.68–12.2% and 0.88–7.84% respectively were comparable to or lower than CVs reported in previous studies evaluating human assays for canine specimens. Evaluated human assays included the Bayer CRP assay (Bayer CRP TIA^,^ Bayer plc, Newbury, Berkshire, United Kingdom: inter- and intra-assay CVs 5.2% - 10.8% and 3% - 10.2% [22]; the Randox CRP assay with human calibrator, Randox Laboratories Ltd., Crumlin, United Kingdom: inter- and intra-assay CVs with human calibrator: 1% - 10% and 18% [23]; the Randox CRP assay with canine calibrator, Randox Laboratories Ltd., Crumlin, United Kingdom: intra-assay CV: 0.7% - 2.1% (own unpublished data); and the Olympus CRP assay, CRP OSR 6147, Olympus Life and Material Science Europe GmbH, Lismeehan, O′Callaghan’s Mills, Ireland 6147: inter- and intra-assay CVs: both <10% [39]). When regarding solely canine species specific CRP assays, inter- and intra-assay CVs obtained for the Gentian CRP test here and in the previous investigation by Hillström et al. (Table 6) were markedly lower than for a commercially available canine CRP ELISA test kit and this can be attributed to the higher variation observed in manual methods (Phase Range canine CRP, Tridelta Development Ltd., Kildare, Republic of Ireland: inter- and intra-assay CVs: 6.9% - 10.1% and 7.5% - 29%) [18]. Muñoz-Prieto et al. showed a similarly low intra-assay CV of 1.0–1.3% for the Gentian CRP test at CRP ranges ≥50 mg/l and an only slightly higher inter-assay CV of 4.1–4.7% (Table 6) still lying below the data of the ELISA test. For a previously developed automated immunoturbidimetric canine CRP assay, intra- and inter-assay CVs <5% and ≤11%, respectively [40] were reported which are comparable to the CVs found in the current and previous studies. The drawback of the previous canine CRP assay, however, was that it was never commercially available. For CRP concentrations >26.5 mg/l, the CVs reported in the previous study evaluating the Gentian Canine CRP Immunoassay on the Abbott Architect c4000 were comparable to our results obtained for the clinically relevant concentration levels B-D) (Table 6). For lower CRP concentrations, intra-assay CVs were not calculated in the previous method validation study by Hillström et al. However, an SD of 0.39 mg/l was obtained for a sample with a CRP concentration of 6.8 mg/l which was consistent with a CV of 5.7% and thus comparable to the inter-assay CV observed here for a similar CRP concentration level. The CV of 5.8% observed by Muñoz-Prieto et al. for a low CRP concentration of ~10 mg/l was also comparable to our and the previous results. Excellent correlation for the Gentian CRP test with the compared assay was shown in the current and both previous studies (Table 6).

When regarding the rationale behind the method validation study performed here, assessment of TE_obs_ is an essential point of each method comparison experiment [29]. In our study, observed CVs and TEs were lower than recommended desirable quality specification published in the addendum of the allowable total error guidelines of the ASVCP [32]. As the quality specifications published by the ASVCP are based on biological variation, they have been considered too stringent for method validation studies [32]. Nevertheless, they were used in this study as no other quality specifications are available for dogs. Even in human studies, quality specifications for CRP are based on biological variation. Interestingly, TE_des_ for CRP reported for people is 56.6% [41] and thus markedly higher than the recommended desirable TE_des_ given for dogs of 29.58% and even slightly higher than the recommended minimally acceptable TE for dogs of 44.37% [32]. Moreover, national recommendations are available for people such as the German RiliBÄK quality specifications (i.e., Guidelines (= RichtLinie “Rili”) of the German Federal Medical Council (= Bundesärztekammer “BÄK”) [42], for which unofficial translations [43] have been performed to allow an international use. Overall, German RiliBÄK quality specifications are most stringent as only deviations of 13.5% are allowed. Interestingly, they are comparable with the recommended TE_opt_ for canine CRP of 14.79% published in the ASVCP guidelines [32]. TE_opt_ being comparable with German RiliBÄK quality specifications has been also observed previously for hematology measurands [44]. For CRP concentrations >58 mg/dl, TE_obs_ was even below these most stringent quality specifications. For lower CRP concentrations close to physiologic values, TE_obs_ was higher which was mainly based on a higher CV. Despite all advantages of the calculation of TE_obs_ (encompassing various sources of error by the inclusion of imprecision and bias) [45], it has to be considered that also the TE_obs_ is not a perfectly objective quality parameter as it is dependent on the reference method used to calculate the bias as was shown before for hematology analyzers [44]. At the moment, there is no consensus about the methodology for bias determination for quality assessments. A high bias does not necessarily indicate a poor assay performance but might be solely induced by differences in the test protocol [46]. Only if the reference method can be considered as a current gold standard, a high bias has to be interpreted as a deficient quality performance. If the quality data of the current study are compared to the bias and TE_obs_ calculated based on data of the previous method comparison with another human based CRP assay [28] at for clinical decisions relevant CRP levels, major differences can be detected which may mainly be due to the different reference method applied (Table 6). Hillström et al. [26] used the same reference assay as was used in our study but did not provide data for an estimation of the TE_obs_. At the moment there is no superior alternative to the assessment of TE_obs_. Even the use of the TE as quality standard is not without limitations as there are several methods of its determination (i.e, derived on experts´ opinion, human quality specifications or biological variation [44]) which might come to different results. For the canine CRP, only a TE derived from biological variation is available, however, it has to be considered that the analytical method and the analyzer initially used for its determination have an impact on the results. Analysis of possible interferences of hemolysis, hyperbilirubinemia and lipemia on the assay performance revealed no interferences in clinically relevant concentrations up to 5 g/l hemoglobin, 800 mg/l bilirubin and 10 g/l soy bean oil (Intralipid). The absence of interference of lipemia and hemolysis with the CRP measurement was also confirmed in the previous study evaluating the species-specific Gentian Canine CRP Immunoassay [26] (Table 6), however, the potential interfering effect of bilirubin was not assessed previously. To the author’s knowledge, this is the first time effects of hyperbilirubinemia are investigated on the Gentian Canine CRP Immunoassay. The lack of interference effects has to be claimed as a major advantage in CRP analysis as associated metabolic states frequently occur in patients with inflammatory and infectious diseases [26]. In contrast, significant interferences for all three substances were noted for a commercial solid phase sandwich immunoassay (Tridelta Phase Range Canine C-reactive Protein Assay; Tridelta Development, Bray, Ireland) although the low magnitude of the differences did not appear of relevance for clinical interpretation of the test [47].

To the authors´ knowledge, the effect of the sample type (heparinized plasma as an alternative to serum) has not been evaluated before for the Gentian Canine CRP Immunoassay. However, there are data available for the dog-specific CRP ELISA (Tridelta Phase Range Canine C-reactive Protein Assay, Tridelta Development, Bray, Ireland). As in our study, there was no major difference (*P* = 0.008) in CRP results measured in heparin plasma or serum, although in contrast to our findings, the results tended to be slightly but insignificantly higher in heparin plasma than in serum [47]. As CRP measurements in heparin plasma showed a similar CV than those performed in serum samples, it can be concluded that both heparin plasma and serum can be used. Due to the small bias, however, follow-up examinations should be ideally performed in the same sample type.

## Conclusion

The results of this study are comparable to the findings observed during the previous evaluation of the canine species-specific CRP test run on different analyzers [26, 28]. The good performance of the test enables its application to several types of large bench top analyzers. However the discrepant findings between the current and previous studies such as the CRP concentration at which a prozone effect occurs, linearity range and LoQ should be specifically evaluated for each analyzer and laboratory performing the test.
